# Xanthine oxidase inhibition for the improvement of long-term outcomes following ischaemic stroke and transient ischaemic attack (XILO-FIST) – Protocol for a randomised double blind placebo-controlled clinical trial

**DOI:** 10.1177/2396987318771426

**Published:** 2018-04-18

**Authors:** Jesse Dawson, Niall Broomfield, Krishna Dani, David A Dickie, Alex Doney, Kirsten Forbes, Graeme Houston, Sharon Kean, Kennedy Lees, Alex McConnachie, Keith W Muir, Terry Quinn, Allan Struthers, Matthew Walters

**Affiliations:** 1 Institute of Cardiovascular and Medical Sciences, College of Medical, Veterinary & Life Sciences, Queen Elizabeth University Hospital, University of Glasgow, Glasgow, UK; 2 Stroke Psychology, West Glasgow Ambulatory Care Hospital, Scotland, UK; 3 Department of Neurology, Institute of Neurological Sciences Glasgow, Queen Elizabeth University Hospital, Glasgow, UK; 4 DD Analytics Cubed Ltd, Bishopton, UK; 5 Medicine Monitoring Unit (MEMO), School of Medicine, University of Dundee. Ninewells Hospital, Dundee, UK; 6 Department of Neuroradiology, Institute of Neurological Sciences, Queen Elizabeth University Hospital, Glasgow, UK; 7 Division of Molecular and Clinical Medicine, School of Medicine, Ninewells Hospital, Dundee, UK; 8 Robertson Centre for Biostatistics, Institute of Health and Wellbeing, College of Medical, Veterinary & Life Sciences, University of Glasgow, Glasgow, UK; 9 Institute of Neuroscience and Psychology, College of Medical, Veterinary & Life Sciences, University of Glasgow, Queen Elizabeth University Hospital, Glasgow, UK

**Keywords:** Stroke, allopurinol, hypertension, white matter hyperintensities

## Abstract

**Background:**

Allopurinol, a xanthine oxidase inhibitor, reduced progression of carotid-intima media thickness and lowered blood pressure in a small clinical trial in people with ischaemic stroke. Xanthine oxidase inhibition for improvement of long-term outcomes following ischaemic stroke and transient ischaemic attack (XILO-FIST) aims to assess the effect of allopurinol treatment on white matter hyperintensity progression and blood pressure after stroke. This paper describes the XILO-FIST protocol.

**Methods:**

XILO-FIST is a multicentre randomised double-blind, placebo-controlled, parallel group clinical trial funded by the British Heart Foundation and the Stroke Association. The trial has been adopted by the Scottish Stroke Research Network and the UK Clinical Research Network. The trial is registered in clinicaltrials.gov (registration number NCT02122718). XILO-FIST will randomise 464 participants, aged greater than 50 years, with ischaemic stroke within the past month, on a 1:1 basis, to two years treatment with allopurinol 300 mg twice daily or placebo. Participants will undergo brain magnetic resonance imaging, cognitive assessment, ambulatory blood pressure monitoring and blood sampling at baseline and after two years treatment. The primary outcome will be white matter hyperintensity progression, measured using the Rotterdam progression scale. Secondary outcomes will include change in white matter hyperintensity volume, mean day-time systolic blood pressure and measures of cognitive function. Up to 100 will undergo additional cardiac magnetic resonance imaging in a sub-study of left ventricular mass.

**Discussion:**

If white matter hyperintensity progression is reduced, allopurinol could be an effective preventative treatment for patients with ischaemic stroke and clinical endpoint studies would be needed. If allopurinol reduces blood pressure after stroke, then it could be used to help patients reach blood pressure targets.

## Background

People who suffer an ischaemic stroke are at risk of cognitive decline and recurrent vascular events.^1–3^ Guidelines recommend use of anti-thrombotic, blood pressure (BP) lowering and lipid lowering agents to reduce subsequent risk. These treatments reduce, but do not eliminate, risk of recurrent stroke and other vascular events and the effect of these treatments on cognitive decline is unclear.^4,5^

Since white matter hyperintensities (WMHs) on brain magnetic resonance imaging (MRI) are associated with higher risk of recurrent stroke and cognitive decline,^6^ it is hypothesised that these represent a biomarker of brain ischaemic injury. WMHs are seen in as many as 90% of people with ischaemic stroke,^7,8^ and the highest degrees of WMH burden are associated with higher rates of stroke, death and cognitive and physical decline.^7,9^ The burden of WMH often increases during longitudinal follow-up and such increases are also associated with increased incident stroke, dementia and cognitive decline.^6^ Thus, treatments that reduce WMH progression could improve several outcomes after stroke including cognition, functional outcome and recurrent stroke.

Allopurinol, a drug commonly prescribed for the prophylaxis of gout, inhibits activity of xanthine oxidase leading to reduction in both serum uric acid (UA) and oxidative stress via reduced superoxide anion production. Higher serum UA is associated with increased risk of stroke,^10^ with adverse outcomes after ischaemic stroke^11,12^ and with vascular cognitive impairment. Allopurinol may deliver benefits independent of UA reduction. In people with history of stroke, allopurinol has been shown to reduce markers of inflammation,^13^ reduce augmentation index, reduce progression of carotid intima-media thickness and lower BP^14^ and has been shown to improve cerebral nitric oxide bioavailability.^15^ Thus, allopurinol has a number of effects on the vasculature, on both small and large cerebral arteries, which may make it an effective drug for stroke prevention. However, trials in people with stroke have typically been small, of short duration, and have not assessed progression of biomarkers of cerebrovascular disease or clinical outcomes.

The xanthine oxidase inhibition for improvement of long-term outcomes following ischaemic stroke and transient ischaemic attack (XILO-FIST) trial is designed to test whether allopurinol reduces the rate of WMH progression and BP in people with recent ischaemic stroke.

## Methods/design

XILO-FIST is a randomised, double-blind, placebo-controlled clinical trial of allopurinol 300 mg twice daily vs. placebo in 464 people with recent ischaemic stroke. The study is being conducted in stroke units in the UK Clinical Research Networks. The trial includes a sub-study of additional cardiac MRI in participants with left ventricular hypertrophy (LVH) and some participants also undergo carotid MRI.

### Trial status

The first participant visit was in May 2015. As of 27 February 2018, the trial was open in 23 sites in the UK. A total of 418 participants were enrolled and 354 participants were randomised (with most of the remainder in the trial run in phase). On average, 12 participants per month are being randomised. One hundred and eighty-eight participants have completed one-year follow up; 57 participants have completed the two-year follow-up. We aim to finish recruitment by May 2018.

### Ethical and regulatory approval

Ethics committee and regulatory approval has been obtained for all participating sites (REC number 14/WS/0113). The trial is being conducted in accordance with local regulations and UK law.

### Inclusion and exclusion criteria

Participants are aged greater than 50 years and have suffered an ischaemic stroke or transient ischaemic attack (TIA) with positive imaging within the past four weeks. Ischaemic stroke and TIA are diagnosed by a stroke specialist. Symptoms must last more than 24 h or for symptoms lasting less than 24 h (TIA), there must be either a relevant diffusion-weighted imaging (DWI) lesion on MRI or a corresponding lesion on CT. The corresponding lesion on CT can include evidence of cerebral small vessel disease. Full inclusion and exclusion criteria are given in Table 1. Participants with evidence of LVH on electrocardiography (ECG, according to the Sokolow-Lyon or Cornell voltage criteria) or on screening echocardiography (defined as posterior or septal wall thickness of >11 mm, or increased left ventricular mass (LVM) defined as baseline LVM index of > 115 g/m^2^ (men) or > 95 g/m^2^ (women), or LVM >162 g (men) or >224 g (women)) and no current atrial fibrillation are eligible for the cardiac sub-study.

**Table 1.** Full inclusion and exclusion criteria.

Inclusion criteria	Exclusion criteria
Ischaemic stroke/ischaemic lesion on brain imaging in relevant anatomical territory in patients with transient ischaemic attack	Modified Rankin scale score of 5
Age greater than 50	Diagnosis of dementia
Consent within one month of stroke	Cognitive impairment deemed sufficient to compromise capacity or comply with the protocol
	Dependent on daily help from others for basic activities prior to stroke
	Significant co-morbidity or frailty likely to cause death within 24 months
	Contra-indication to or indication for administration of allopurinol
	Concurrent azathioprine, 6-mercaptopurine therapy, other cytotoxic therapies, cyclosporin, theophylline and didanosine
	Significant hepatic impairment
	eGFR < 30 ml/min
	Contraindication to MRI scanning
	Women of childbearing potential
	Prisoners
	Active participation in another CTIMP or device trial or participation within the past month
	eGFR < 60 and of Korean, Han Chinese or Thai descent

MRI: magnetic resonance imaging; eGFR: estimated glomerular filtration rate; CTIMP: Clinical trial of investigational medicinal product.

### Participant identification and recruitment

Potential participants are identified during in-patient stay in an acute stroke unit or in a cerebrovascular out-patient clinic. Eligibility is confirmed by a medically qualified investigator and participants must give their own informed consent.

### Study schedule

The study comprises a four-week run-in phase and a 104-week treatment phase. The detailed participant schedule is shown in Table 2.

**Table 2.** Participant schedule.

Activity	Run-in phase		Treatment phase						
	Day 0	Week 4	Week 0	Week 4	Week 13	Week 26	Week 52	Week 78	Week 104
Review eligibility	✓	✓							
Informed consent	✓								
Optimise preventative therapy	✓								
Clinical evaluation^a^	✓	✓		✓	✓	✓	✓		✓
Safety blood tests^b^	✓	✓		✓	✓	✓	✓		✓
Blood for uric acid level		✓							✓
Blood/urine for biobanking		✓							✓
ECG		✓							✓
Echocardiography		✓							
Determine cardiac sub-study eligibility		✓							
MRI brain (± carotid MRI)		✓							✓
ABPM		✓		✓					✓
Cardiac MRI,^c^ n=100		✓							✓
Detailed cognitive function evaluation		✓							✓
Assessment of run in completion		✓							
Randomisation			✓						
Dispense			✓	✓	✓	✓	✓	✓	
Return/count IMP				✓	✓	✓	✓	✓	✓
Adverse event review			✓	✓	✓	✓	✓	✓	✓

Note that the run in week 4 and treatment phase week 0 visits can take place concurrently. Participants will also be contacted by telephone at week 105, which marks the end of the study.

IMP: investigational medicinal product; ABPM: ambulatory blood pressure monitoring; ECG: electrocardiography; MRI: magnetic resonance imaging.

^a^Measures of stroke severity at week 4, modified Rankin scale score and MoCA at week 52 and blood pressure at all visits except week 78 and weight at week 104).

^b^FBC, U + E, LFTs.

^c^Sub-study eligible participants only.

### Run-in phase

The run-in phase comprises an enrolment visit on day 0 and a baseline assessment visit at four weeks. In order to successfully complete the run-in phase, participants must have had a medication review, completed baseline data collection and completed brain MRI. Those who do not successfully complete this phase are classed as screen failures.

The baseline assessment visit includes a clinical evaluation (measurement of brachial sphygmomanometer BP), blood tests for safety and biobanking (including a blood test for serum UA to be centrally analysed), a urine sample for biobanking, ambulatory blood pressure monitoring (ABPM), ECG, brain MRI, assessment of cognitive function and assessment of eligibility for the cardiac sub-study (in participating sites only). In sites with experience of carotid plaque MRI, carotid MRI will be performed after the brain MRI scan.

Randomisation is performed following successful completion of the run-in phase. Study medication then starts.

### The treatment phase

Week 4 visit: Brachial sphygmomanometer BP is measured, safety bloods are taken, participants are asked regarding adverse events (AEs) and an ABPM is performed. Dose of study drug is increased at this visit if the criteria are met for dose escalation.

Week 13/26/52/78 visit: Brachial BP is measured, safety bloods are taken (except at week 78), participants are asked regarding AEs and an ABPM is performed. A Montreal Cognitive Assessment (MoCA) is also performed at week 52.

Week 104 visit: Brachial BP is measured, safety bloods and bloods for biobanking are taken, participants are asked regarding AEs and an ABPM is performed. Brain MRI and cognitive testing are repeated. Participants in the cardiac sub-study undergo an additional cardiac MRI. A further assessment for serious AEs (SAEs) is made at week 105 by telephone.

### Randomisation

Participants are randomised (1:1) to receive either allopurinol or placebo orally for 104 weeks. Randomisation codes are stored securely on the Robertson Centre for Biostatistics (RCB, University of Glasgow) network, with restricted access. Eighty per cent of participants are allocated to treatments by a minimisation algorithm which includes presence of WMH at baseline and sub-study eligibility as minimisation factors, Randomisation carried out via the study web portal. A telephone interactive voice response system is available as a backup.

### Intervention

Participants receive either allopurinol 300 mg or placebo twice daily for two years. During the first four weeks, a single 300 mg daily dose of allopurinol or placebo is taken. All participants then undergo dose titration to allopurinol 300 mg twice daily or placebo unless creatinine clearance (estimated via estimated glomerular filtration rate (eGFR)) is < 60 mL/min where once daily dosing is continued. Dose modification (a reduction from 300 mg twice daily to 300 mg once daily) occurs if renal function declines (to an eGFR of < 50 mL/min) or in the event of side effects. Dosing is stopped if renal function declines to an eGFR of < 30 mL/min. After the 104-week assessment, treatment with study medication stops.

### Blinding

The study is double blind. An identical placebo is used. Changes in UA levels could compromise allocation concealment and these are not to be checked during the study. Unblinding should only occur in emergency situations where knowledge of the investigational product assignment is essential for the care of the participant. All investigators have received training in unblinding procedures.

### Brain and carotid MRI

Brain MRI is performed using 1.5 or 3T MRI, and in each individual, the same scanner should be used for baseline and follow-up. Study sequences include T1-weighted imaging, T2-weighted imaging and T2 fluid attenuation inversion recovery (FLAIR), DWI and susceptibility weighted imaging. Isotropic T1, T2 and FLAIR imaging will be performed where possible (Table 3). Carotid MRI imaging includes time of flight carotid angiography, black blood T1, black blood T2 and black blood proton density imaging of the carotid arteries.

**Table 3.** Sequence parameters used for brain and carotid imaging in co-ordinating centre and on Siemens PRISMA 3T system.

Scan	Sequence	Orientation	TE	TR	TI	Slice Thickness	Slice gap	Matrix	FOV	Slice number	Total time
Brain											
T1	TFL	SAG	1.85	2000	900	1.0	50%	256 × 100	255	176	4.4
T2	SPC	TRA	404	3000	–	0.9	–	256 × 100	230	176	5.32
FLAIR	SPCIR	SAG	397	5000	1800	1.0	–	256 × 100	255	160	4.02
DWI	RESOLVE	TRA	62	4100	–	4	30%	224 × 100	220	27	3.55
SWI	SWI_r	TRA	20	24	–	1.5	20%	256 × 95	230	96	4.45
DTI	EPSE	TRA	95	3600	–	4	30%	128 × 100	230	30	2.51
ASL	EPFID	TRA	11	3500	–	6	16%	64 × 100	255	20	6.06
Carotids											
TOF	FL_r	TRA	3.11	20	–	1.0	–	384 × 75	200	32/3 slabs	2.57
T1	tse	TRA	17	740	–	2.0	50–200%	256 × 100%	140	5 to 11	2.37
T2	tse	TRA	79	740	–	2.0	50–200%	192 × 100%	140	5–11	1.51
PD	tse	TRA	16	740	–	2.0	50–200%	192 × 100%	140	5–11	1.51

FLAIR: fluid attenuation inversion recovery; DWI: diffusion weighted imaging; SWI: susceptibility weighted imaging; DTI: diffusion tensor imaging. ASL: arterial spin labelling. FOV: field of view, TRA: transverse; SAG: sagittal; TOF: time of flight; PD: proton density; TE: echo time; TR: repitition time; TI: inversion time.

### Assessment of WMH

The STRIVE recommendations are followed during image review.^16^ WMH of presumed vascular origin are defined as hyperintense lesions on T2-FLAIR and can appear as isointense or hypointense (although often not as hypointense as CSF) on T1-weighted sequences. All scans are reviewed blinded to treatment allocation.

A Fazekas and Scheltens scales is assigned.^17,18^ The Rotterdam progression score (RPS) and Schmidt’s progression score are calculated by simultaneous review of the baseline and two-year scans.^19,20^ All visual rating scales are assessed independently by two trained observers. Where there is any level of disagreement on a score, that score will be reviewed by at least two raters and a consensus score applied.

Volumetric assessment of WMH volume will also be performed. The first step in automated extraction of WMH volumes is to estimate the white matter area in each subject using atlas-based segmentation.^21^ A probability map of white matter created from 313 volunteers aged 18–96 years is used,^22^ and this map is registered to each subject using non-linear (diffeomorphic) registration to provide an initial estimate of white matter in each subject.^21^ Hyperintense outliers are identified on T2 FLAIR by transforming each voxel to a standard (z) score. Voxels with z ≥ 1.5 and within the estimated white matter area are initially defined as WMH. Final WMH estimates are defined by 3D Gaussian smoothing to reduce noise and account for partial volumes around WMH edges. Automatic WMH estimates are visually checked and infarcts masked by a trained image analyst following STRIVE guidelines.^16^ Normal-appearing tissues including cortical grey matter, sub cortical grey matter, cerebral white matter and supratentorial cerebrospinal fluid are segmented using population-specific tissue probability maps, within-patient T1 intensity data, and adjoining voxel data.^23,24^ Normal-appearing tissue segmentations are checked and edited in the same manner as WMH.

### Cardiac sub-study

Participants in the sub-study undergo additional cardiac 3T MRI at baseline and two years.

### ABPM

Twenty four-hour ABPM is performed at baseline, week 4 and week 104 unless contraindicated. A Spacelabs Ultralight Ambulatory Blood Pressure Monitor is used. This is set to take readings every 30 min during daytime (08:00 h–21:59 h) and every 60 min during night-time (22:00 h–07:59 h). ABPM data will not be performed in some participants with significant arm weakness due to safety concerns.

### Cognitive and quality of life measures

The assessment of pre-stroke cognitive impairment uses the 16-item IQCODE.^25^ A score of 3.6 or greater is used as threshold to define probable pre-stroke dementia.

A comprehensive cognitive examination will be performed at baseline and at the two-year follow-up. The battery comprises: the MoCA; Animal Naming test of semantic fluency; Controlled Oral Word Association Test; Letter Digit Coding Test; Hopkins Verbal Learning Test; Centre for Epidemiological Studies – Depression Scale (CES-D); neuropsychiatric inventory questionnaire version (final follow-up visit only) and a trail making test. The EQ-5D and the Stroke Impact Scale Short Form^26^ are also measured.

The battery is administered by a trained assessor, scored to pre-specified marking sheets and are conducted in a standardised fashion. Participants are free to take breaks as needed. If participants are unable to complete the full battery, the assessor prioritises the MoCA and CES-D. Details of each of these scales are given in a detailed instruction booklet, which includes instructions for administering each assessment.

### Safety blood tests and pharmacovigilance

Safety blood tests including a full blood count, urea and electrolytes and liver function tests are checked at all study visits with the exception of the week 78 visit. Blood for serum UA levels is obtained at the end of the run-in phase (baseline visit) and at week 104 during the treatment phase. Serum UA levels will be measured centrally.

Predictable side effects of the investigation medicinal product (IMP) used in this trial (allopurinol) are referred to in the summary of medicinal product characteristics (SmPC). All SAEs occurring within the first 13 weeks of the treatment phase will be recorded and reported to the Sponsor. Thereafter and up to 30 days after completing the study, unexpected SAEs, suspected unexpected serious adverse reactions and events of special interest that meet criteria for an SAE will be reported.

### Criteria for stopping study medication and study withdrawal

Participants developing a rash, fever, liver dysfunction (defined as bilirubin or transaminase levels increasing to three times the ULN), renal dysfunction (defined as a drop in eGFR to below 30 mL/min), eosinophilia (defined as an eosinophil count of > 0.45 × 10^9^ or a fall in haemoglobin below 10 g/dL or neutrophil count of <1.5 × 10^9^ on any blood sample of a platelet count of <50 × 10^9^ that is not due to clumping cease taking study medication immediately and permanently if no alternative cause is found. We will not recruit additional participants to replace those who stop treatment and participants who withdraw from treatment will be asked to remain under follow-up.

### Study outcomes

The primary outcome is WMH progression measured using the RPS. Secondary outcomes and exploratory outcomes are shown in Table 4. The primary outcome in the cardiac sub-study is LVM index.

**Table 4.** Primary, secondary and exploratory outcomes.

Primary outcome	Secondary outcomes	Exploratory outcomes
Rotterdam progression scale	Change in mean day-time systolic BP at one month	Fazekas’ score
	Change in mean day-time diastolic BP at one month	Schelten’s scale score
	Schmidt’s progression score	Measures of BP variability
	WMH volume at two years	One month mean day-time diastolic blood pressure
	Fazekas’ score at two years	Two-year mean day-time diastolic BP
	Schelten’s scale score at two years	Clinic brachial BP
	New brain infarction on MRI	Incident atrial fibrillation
	Rotterdam progression score with those who die/become too frail to undergo repeat imaging assigned worst score	Recurrent stroke
	MoCA score	Recurrent myocardial infarction, stroke or cardiac death
	Change in mean day-time systolic BP at two years	Hospitalisation for, or incident heart failure
	Change in mean day-time diastolic BP at two years	Incident dementia
		Mortality
		Animal naming test
		Controlled word association test
		Hopkins verbal learning test
		Trail making test
		Quality of life (EQ-5D, SS-QOL)
		Modified Rankin scale score

BP: blood pressure; MoCA: Montreal cognitive assessment.

### Statistical analysis

Full details of all statistical issues and planned statistical analyses will be specified in a separate statistical analysis plan which will be agreed before database lock and unblinding of treatment codes to the study statisticians.

All data will be summarised overall and by treatment group. Efficacy analyses will be carried out according to the intention-to-treat (ITT) principle, that is, in relation to randomised treatment allocation, regardless of treatments actually received. The population for ITT analyses will be all validly randomised participants, who do not have any major protocol violations. Additional analyses will be carried out using a per-protocol (PP) population, consisting of those members of the ITT population who remain on treatment as randomised and do not have any minor protocol violations. All protocol violations will be assessed and classified as major or minor, prior to database lock.

Unadjusted between-group comparisons of continuous and ordinal outcomes will be made using t-tests or Wilcoxon-Mann-Whitney tests as appropriate; categorical outcomes will be compared using Fisher’s exact tests. Adjusted analyses will use regression models, adjusted for variables used in the minimisation algorithm. Distributional assumptions will be assessed visually and where necessary, outcomes will be transformed, or a generalised linear model will be used with appropriate link and variance function. The model to be used for each outcome will be decided and documented prior to database lock. Treatment effect estimates will be reported with 95% confidence intervals and p-values.

Analysis of primary outcome – WMH progression is not expected to follow a normal distribution. Unadjusted comparison of treatment groups will use a Wilcoxon-Mann-Whitney test. A generalised linear regression model, with appropriate link and variance function, will be used to model WMH progression in relation to treatment and variables used in the minimisation algorithm. A further model will be fitted adjusting for other baseline characteristics found to be associated with WMH progression during blinded analyses, prior to database lock.

This model will be extended to assess the mediating effects of other study outcomes, such as changes in BP and UA post-randomisation.

Analysis of secondary outcomes – secondary efficacy outcomes will be analysed using appropriate two-sample tests followed by regression analyses to estimate between-group differences adjusted for variables used in the minimisation algorithm. For outcome measures recorded at baseline as well as at follow-up, regression models will be adjusted for the baseline value. For endpoints measured at several time points, each time point will first be analysed separately, and then a repeated measures model will be applied to model measurements at all time points simultaneously.

Planned subgroup analyses – the moderating effects of baseline UA level in the primary analysis will be assessed through use of interaction terms in the model. These methods will also be used to investigate the moderating effects of other baseline characteristics in an exploratory manner.

### Sample size

We assumed that 90% of participants would have evidence of WMH at baseline and that, over two years, approximately 64% would progress by one point or more on the RPS and that the mean progression score in the placebo group will be 1.293. This and the progression rate seen with a 30% reduction in WMH progression score are shown in Table 3. Based on a Wilcoxon-Mann-Whitney test, a sample size of 192 participants per group would give 80% power to detect this difference at a 5% significance level (nQuery Advisor® v7.0). This treatment effect is substantially less than the 80% relative reduction seen in the PROGRESS MRI study.^17^

Although we will minimise loss to clinical follow-up (completed for all except one participant in our pilot study), we have increased sample size to account for a 10% drop out rate seen in our pilot study and by an additional small amount to account for the fact that those with no WMH at baseline may progress at a lower rate. We will thus randomise 232 participants per group (10% drop outs will give data on 209 participants giving a further 17 participants (8%) per group to ensure sufficient power).

Other sample size calculations were based on two-sample t-tests. For ABPM, 101 participants per group will be required to give 80% power at a 5% significance level to verify the 3.3 mmHg reduction in systolic BP (SBP) seen in the recent meta-analysis^18^ (assumed SD of change in SBP 8.3). For the cardiac sub study, 25 participants per group would give 80% power to detect a 3.7 g difference in LV mass (assumed standard deviation 4.6). A 3.86 g difference was seen in a recent trial of allopurinol use in patients with type 2 diabetes.

### Trial and data management

All study data will be held in the study Robertson Centre for Biostatistics, University of Glasgow, part of the UKCRC-registered Glasgow Clinical Trial Unit (number 16). A secure and restricted electronic data capture system (electronic case report form) will be used.

A Trial Management Group (TMG) meets regularly during the study.

#### Trial steering and data monitoring committees

A Trial Steering Committee meets at least annually and comprises an independent chair, three other independent members, a participant and/or a carer representative, the chief investigator and statistician. An independent data monitoring committee (IDMC) meets at least annually. This comprises of four independent members. Only the IDMC has access to unblinded outcome data before the trial ends.

### Summary and conclusion

XILO-FIST is designed to test whether allopurinol reduces the rate of WMH progression and BP in participants with recent ischaemic stroke. The hypothesis underlying XILO-FIST is that UA reduction and xanthine oxidase inhibition will improve vascular health and lower BP leading to a reduction in progression of WMH progression on MRI. If WMH progression is reduced, allopurinol could be an effective preventative treatment for people with ischaemic stroke and clinical endpoint studies would be needed. If allopurinol reduces BP after stroke, then it could be used to help people reach BP targets.

## Authors’ note

A writing committee will be convened and be responsible for writing all abstracts and manuscripts for publication. The writing committee will consist of the applicants and TSC chair and will be responsible for approving content and dissemination. Data will be shared after the trial is complete, all planned analysis have been completed and subject to data sharing agreements with the University of Glasgow.

TSC members: Prof Philip Bath (Chair), Ms Shannon Amolis, Prof Jesse Dawson, Dr Kirsten Forbes, Dr Richard Francis, Prof Mark Kearney, Prof Kennedy Lees, Dr Alex McConnachie, Dr Marc Randall, Mr Charles Ross (Lay Member), and Prof Allan Struthers.

IDMC members: Prof Peter Sandercock (Chair), Prof Gary Ford, Dr Chris Sutton, and Dr William Whiteley.
